# Items

**Published:** 1881-10

**Authors:** 


### Items.

SUGGESTED PROGRESS IN MEDICAL JURISPRUDENCE. — The *St. Louis Medical and Surgical Journal*, recently contained the following suggestions :

1. In certain cases a jury of medical men should be obtained if possible.

2. Medical experts should be summoned and paid by the State, the experts acting as associate judges, assisting the judge to get at the truth.

3. Certain cases might be referred to a commission, fashioned somewhat like the present commissions in lunacy.

4. Experts called by the court to testify, should be paid. In the State of Indiana, the Supreme Court has decided, "that, under the constitution, the State has no right to take a man's particular services without compensation, and that the giving of expert medical testimony is a particular service within the meaning of the constitution."

5. The office of coroner, with the system of coroner's juries now prevailing, should be abolished, as suggested by the President of the New York State Society in 1879, and the system in vogue in Massachusetts be adopted.

4. Life Insurance companies have accumulated a mass of information relative to causes of death, the bearings of certain conditions upon the question of insanity, or of longevity, facts proving or disproving the commission of suicide, etc., etc. At present this information, so useful in the court room, is inaccessible to the majority of physicians. Is it not possible for "medical examiners," individually or conjointly, to bring this material before this society for our edification and learning.

7. Cannot members in good standing in our society be protected against irregulars by enforcing the regulation law? If men are to be permitted to practice by license, is a license granted in some other county good in this county?

8. The few instances in which examinations of supposed invalids have been ordered by the court to be held in the presence of medical men, acting for the court, as "referees and conductors," have been appreciated by the profession interested on both sides of the case: (Proc. Med. Soc. Co. of Kings.)

CRIMINAL LUNATICS.—To place insane convicts in the same institutions, and the same apartments, with insane patients from the best families of our best citizens, is inconsistent with every dictate of propriety. If a wing for insane convicts cannot be provided in connection with the State Prison, a separate building sufficiently secure and suitable for the purpose, should be erected for their accommodation: (Report of the Managers of the New Jersey State Asylum.) The medical superintendent of the same, points out that an ordinary lunatic hospital is not constructed in such a manner as to render it suitable for the detention of persons who add determined criminal propensities to insanity, and that it would destroy its hospital character, to adapt it to that class; and he pleads for the erection of a separate asylum, meeting the requirements of security, and so organized as to supply humane treatment to the really insane, without creating any incentive to convicts to feign insanity.

MENTAL STATE OF GUITEAU.—If the reports are substantially correct, that Guiteau's uncle and two cousins were insane; that he was as a boy ill-balanced, and not to be depended upon; as a youth, irritable, full of vagaries, and without power of concentration in steady work; as a man, possessed with constantly varying extravagant delusions, for which there was no sort of reasonable foundation, with a mind always vacillating between several impracticable projects; that he made a homicidal attack upon his sister with an ax several years ago, without any real provocation; that he has pursued several women with wild plans of marriage; that he has threatened various persons upon trivial grounds; and finally that he supposed he should do the country a great service, and obey God's direct command, if he could succeed in murdering the head of the government; or even if dissapointment, revenge, political illusions, furnished the chief motives for his act, there can be no kind of doubt that he is a lunatic with delusional insanity of so long standing as to have produced very considerable dementia in a mind of originally bad quality.

The attempt to kill President Garfield differs only in unimportant details from events with which we are made almost daily familiar through the newspapers, and which are sure to occur from time to time so long as irresponsible, insane persons, are allowed to be at liberty. Although there can never be any satisfactory definition of insanity, any absolute criterion of responsibility, or any human means of determining, in doubtful cases, how far the possible self-control had been exercised, it is difficult to avoid the conclusion that all criminals, sane or insane, should be treated from the standpoint of the safety and good of society. The insane asylum is not a safe enough place of custody for the insane of the criminal class; we really need separate criminal lunatic asylums.

It rests with us to estimate the mental condition of the criminal insane, from as nearly a judicial point of view as is possible; it remains for the legislatures and courts, and not for us, to decide what the protection of society demands: (Boston Med. and Surg. Journal.)

CADAVERIC ALKALOIDS.—The very serious question, from the medico-legal point of view, of the spontaneous development of cadaveric alkaloids and their toxic power, was first raised by M. Selmi, of Bologna. After many vicissitudes of alternate favor and discredit, this subject has been revived in France by the researches of MM. Brouardel and Boutmy; in Germany by those of M. Henseman, and in Italy by the *résumé* of all the information on the subject recently published by the original discoverer, M. Selmi. The activity of some of these substances is in no degree inferior to that of the strongest poisons. There are many distinct ptomaïnes, each showing special chemical and physiological characteristics: and they noted the following fact, of which the importance is clear, that one of these ptomaïnes, extracted from a corpse which had been eighteen months in the Seine, had all the properties of veratrine. Huseman has taken up the study of ptomaïnes from another point of view. He has felt doubtful whether these substances may not account for the poisoning by alimentary substances and putrid infections. The researches of Hoppe-Seyler, Schmidt, Schmideberg, and Panum, have shown, in alimentary substances in a state of putrefaction, the presenc of alkaloids analogous to ptomaïnes. Huseman concluded that, as a matter of fact, the majority of the accidents observed in persons who had partaken of food in a state of commencing putrefaction, are to be imputed to these alkaloids, which are of simiiiar constitution to ptomaïnes: (Medical News.)

A REUTER'S telegram announces that a whole boat-load of persons out for a picnic at Warrensburg, U. S. A., who partook of lemonade, were poisoned with it; eight of them are reported in the telegram to be dead, and one hundred others in a critical condition. No indication was given of the nature of the poison, and it was very probably a mineral poison. The sources of poison in drinks of the kind are twofold, being either due to incidental impurities in the ingredients—whether acids or salts—or to the apparatus in which they are made, and the consequent production of symptoms of lead poisoning. Some time since, when public attention was directed toward the subject considerably, more or less important quantities of lead were found to be frequently present in the artificial lemonade of commerce, and in other artificial aërated drinks. It is probable in this case, however, that some much more considerable accident had occurred, and that the deaths were due to the substitution of a toxic material for that which was intended. Organic and mineral impurities are not the worst enemies which the consumers of artificial waters have to fear when these waters are not prepared from sources of the utmost purity. There is a well known case in which a shooting party, who partook freely of artificial mineral waters, were, on separating, attacked in their different homes by typhoid fever, traced by the medical officer of health to the organic impurity of the drinks which they had been consuming.

The occurrence of startling incidents such as these serve especially to direct attention to the advantages of an absolutely pure mineral water. One of the first circumstances which gave a great impulse to the popularity which Apollinaris Water has now so largely achieved and so firmly established, was the public statement of an eminent hospital physician in London, a Fellow of the Royal Society, in which he described how he had himself been suffering for many months from chronic symptoms of poisoning, of which he was unable to determine the source until, having identified the cause of his disease as being lead poisoning, he succeeded in tracing it to his habitual use of soda water in syphons. The soda water had taken up enough lead from the fittings of the syphons in which it was enclosed, frequently for long periods before use, to produce the symptoms of chronic lead poisoning.—*London Sanitary Record.*

STEPHENSON COUNTY SOCIETY OF PHYSICIANS AND SURGEONS.—Officers: *President*, L. G. Voigt, M.D.; *Vice President*, B. H. Bradshaw, M.D; *Secretary and Treasurer*, K. T. Stabeck, M.D. *Standing Committees:*—Surgery—B. T. Buckley, M.D.; L. A. Mease, M.D. Obstetrics—B. H. Bradshaw, M.D.; T. L. Carey, M.D. Practice of Medicine—F. W. Hance, M.D.; E. A. Carpenter, M.D. Materia Medica—K. S. Marlin, M.D.; F. A. Darling, M.D. Physiology and Pathology—C. M. Hillebrand, M.D.; C. Brundage, M.D. Dermatology—L. Stoskopf, M.D.; F. C. Winslow, M.D. Publication—B. T. Buckley, M.D.; K. T. Stabeck, M.D.; T. L. Carey, M.D.

The Society is regulated by the code of ethics of the American Medical Association, and the State Medical Society of Illinois. Regular meetings are held quarterly.

